# Magnetic Resonance Imaging Evaluation of Azygos Vein Deviation and Risk of Pedicle Screw Penetration in Thoracic Adolescent Idiopathic Scoliosis

**DOI:** 10.7759/cureus.117170

**Published:** 2026-09-29

**Authors:** Goutham B Chand, Binoy Sidhardhan, Ashok Ramakrishnan, Siddharth Babu Chand, Gayathry Santhosh, Meenakshi K G

**Affiliations:** 1 Department of Orthopaedics and Traumatology, Government Medical College, Thiruvananthapuram, Thiruvananthapuram, IND; 2 Department of Orthopaedics, Government Medical College, Thiruvananthapuram, Thiruvananthapuram, IND; 3 Orthopaedic Surgery, Bangalore Medical College and Research Institute, Bengaluru, IND; 4 Orthopaedic Surgery, Sree Narayana Institute of Medical Sciences, Kochi, IND; 5 Department of Trauma and Orthopaedics, Northern Care Alliance NHS Foundation Trust, Manchester, GBR; 6 General Surgery, Dr. D. Y. Patil Medical College, Hospital &amp; Research Center, Map of Dr. D. Y. Patil Vidyapeeth, Pune Dr. D. Y. Patil Vidyapeeth, Pimpri, Pune, Pune, IND

**Keywords:** adolescent idiopathic scoliosis, anatomical variation, azygos vein, mri, pedicle screw fixation, preoperative planning, spinal deformity, surgical risk assessment, thoracic spine, vertebral angle deviation

## Abstract

Objective: The primary objective was to compare the azygos vein-vertebral angle (AVVA) at T7-T10 in patients with thoracic adolescent idiopathic scoliosis (AIS) with previously published reference values. Secondary objectives were to determine the frequency of virtual pedicle screw trajectory-azygos vein intersection, compare AVVA between the concave and convex sides of the curve, and assess the association between AVVA and the main thoracic Cobb angle.

Methodology: This was a cross-sectional study, which was carried out in the Department of Orthopaedics, Government Medical College, Thiruvananthapuram, over a period of one year. There were no normal individuals who did not have scoliosis in this research. To obtain good visibility of the azygos vein, images of the axial MRI at the T7-T10 levels, with the mid-vertebral body in the centre, were acquired. The AVVA is the angle formed between a line through the centre of the vertebral body perpendicular to the longitudinal axis of the vertebra and a line running through the centre of the vertebral body to the azygos vein. The azygos vein was in contact with the left at the AVVA 0^o^, and with the right at 180^o^. The virtual pathway of a pedicle screw was drawn on the pedicle axis on both sides, starting at the posterior cortical entrance point of the pedicle to the anterior vertebral cortex. This path was believed to be at risk of being damaged by the implantation of pedicle screws if this path was crossed. The proportion of azygos veins perceived to be at risk was calculated on both sides in relation to each patient.

Results: Among the 41 subjects with thoracic AIS, a statistically significant deviation of the AVVA compared with previously reported normal reference values from the literature was observed at the T7, T8, T9, and T10 levels using a one-sample t-test (p = 0.00). These findings indicate substantial displacement of the azygos vein from its normal position in these patients. Risk assessment of pedicle screw penetration was performed using an independent t-test.

Conclusion: The study demonstrates significant deviation of the azygos vein from its normal position in patients with thoracic AIS. This altered positioning increases the risk of injury to the azygos vein during pedicle screw fixation.

## Introduction

According to the Scoliosis Research Society, scoliosis is characterized by the Cobb method of measuring a lateral curvature of the spine on a standing radiograph exceeding 10° [1]. The etiology of idiopathic scoliosis, a structural disfigurement, remains unclear. Radiography and clinical assessment normally identify secondary causes of scoliosis [2]. The majority of the cases of idiopathic scoliosis might be classified as adolescent idiopathic scoliosis (AIS) and occur between the ages of 10 and skeletal maturity.

Age at diagnosis is used to classify idiopathic scoliosis. Less than 1% of occurrences of infantile scoliosis occur before the age of three. A diagnosis of juvenile scoliosis is made between the ages of three and 10. Despite the fact that scoliosis has always been identified clinically as a lateral curvature of the spine, the deformity is more complex, and accurate characterization requires measurements and terminology in three dimensions [3].

There are three groups of idiopathic scoliosis that are classified according to three distinct growth phases: infantile (below age three years), juvenile (ages five to eight years), and adolescent (at age 10 to cessation of growth) [4]. Idiopathic scoliosis is found in about 2-2.5% of adolescents with a Cobb angle of 10° or more [5]. It is only about 0.25% of the cases that proceed to a stage where treatment by a doctor is required. Non-surgical therapy aims to stop the development of curves and reduce the need for surgery. 

Non-surgical management aims to limit curve progression and reduce the likelihood of progression to a surgical threshold in appropriately selected patients. Bracing is an established non-operative treatment for skeletally immature patients with curves within the range for which bracing is indicated. At the same time, physiotherapy and scoliosis-specific exercises may be used as adjunctive approaches. Surgical treatment is generally considered for patients with progressive or larger curves depending on skeletal maturity, curve characteristics, and clinical context [6-8].

The thoracic pedicle screw has increasingly become popular as an alternative to hook- or wire-based instrumentation in the surgical management of AIS. It makes larger amounts of deformity correction, shorter lengths of the fusion, and reduced corrective losses after surgery possible [9]. However, the translation or rotation of scoliotic vertebrae might change adjacent essential structures, decreasing the safe zones for instrumentation, making thoracic pedicle screw placement difficult in AIS patients.

The primary objective of this study was to compare the azygos vein-vertebral angle (AVVA) at T7-T10 in patients with thoracic AIS with previously published reference values. The secondary objectives were to determine the frequency of virtual pedicle screw trajectory-azygos vein intersection, compare AVVA between the concave and convex sides of the curve, and assess the association between AVVA and the main thoracic Cobb angle.

## Materials and methods

Study design and setting

This cross-sectional study was carried out over the course of one year, from October 20, 2023, to October 19, 2024. After receiving approval from the Institutional Ethics Committee (approval no. 16/01/2023/MCT, dated 20-10-2023), this cross-sectional study was carried out over the course of a year at the Department of Orthopaedics at Government Medical College, Thiruvananthapuram. Patients with thoracic AIS, as determined by radiological investigation and clinical examination, made up the study population. No normal subjects without scoliosis were included in this study.

Study population and eligibility criteria

Patients aged 10-19 years with a main thoracic scoliosis curve and a Cobb angle of 40°-75° were included. Only patients with thoracic AIS confirmed radiographically were eligible. Patients with a history of previous thoracic or spinal surgery, or congenital cardiac or thoracic abnormalities affecting thoracic anatomy, were excluded.

Sample size calculation

The sample size was calculated using the following formula:

\[ n = \frac{Z_{1-\alpha/2}^2 \sigma^2}{d^2}, \]

where $Z_{1-\alpha/2} = 1.96$ at a 5% significance level.

Values of the standard deviation (σ = 11.7) and precision (d) were derived from a previous study by Jun Jiang et al. [10]. The calculated sample size was 36. To account for potential dropouts and ensure adequate representation, 41 patients were enrolled.

Sampling technique and study variables

Eligible patients attending the Orthopaedic Outpatient Department who provided informed consent were consecutively enrolled until the target sample size was achieved. Recorded demographic and clinical variables included age, sex, main thoracic Cobb angle, and AVVA.

Data collection tool and technique

All eligible patients underwent standing anteroposterior or posteroanterior whole-spine radiographs to assess spinal curvature. The main thoracic Cobb angle was measured using the standard Cobb method by identifying the superior endplate of the upper end vertebra and the inferior endplate of the lower end vertebra of the main thoracic curve. The corresponding endplate lines were used to determine the Cobb angle. 

Preoperative MRI was subsequently evaluated to assess the relationship between the thoracic vertebrae and the azygos vein. Axial MRI images at T7, T8, T9, and T10 were selected for analysis. The AVVA measurement technique was adapted from the method described by Jiang et al. [10]. On each axial image, the midpoint of the vertebral body was identified, and the longitudinal axis of the vertebral body was established. A reference line perpendicular to the longitudinal axis of the vertebral body was drawn through its midpoint. A second line was drawn from the vertebral body midpoint to the centre of the azygos vein. The angle formed between these two lines was defined as the AVVA.

The MRI measurements were performed using PACS (Picture Archiving and Communication System) Client software. Each AVVA measurement was performed twice, and the mean of the two measurements was used in statistical analyses to reduce the effect of measurement variability.

To assess the potential anatomical relationship between the azygos vein and the intended pedicle screw trajectory, a virtual trajectory was constructed bilaterally at each vertebral level from T7 to T10. The trajectory began in the posterior cortical region of the pedicle and extended anteriorly toward the anterior vertebral cortex. The same trajectory definition was applied consistently across the evaluated levels. A trajectory-azygos vein intersection was recorded when the simulated trajectory crossed the course of the azygos vein on the axial MRI image. Such an intersection was classified as a potential anatomical trajectory-vein conflict and not as confirmed vascular injury.

Because the study used a virtual trajectory rather than actual screw implantation, screw diameter and screw length were not independently assigned or evaluated as study variables. Similarly, the virtual assessment did not incorporate intraoperative trajectory alterations, instrumentation-specific adjustments, or surgeon-dependent modifications. The purpose of the simulation was to assess the anatomical relationship between the planned pedicle trajectory and the azygos vein on preoperative MRI.

Information regarding previous conservative treatment, including the type and duration of bracing or other non-operative treatment before surgical evaluation, was not systematically recorded as a study variable and was therefore not included in the analysis.

Statistical analysis

Data were entered into Microsoft Excel (Microsoft Corp., USA) and analysed using IBM SPSS Statistics for Windows, Version 25.0 (IBM Corp., Armonk, NY). AVVA values in AIS patients were compared with MRI values from normal subjects using a one-sample t-test. Pearson correlation analysis assessed the relationship between AVVA and the main thoracic Cobb angle in AIS patients. Statistical significance was set at p < 0.05.

## Results

A total of 41 patients with thoracic AIS were included. Of these, 16 (39.02%) were male, and 25 (60.98%) were female. The patients were aged 11 to 18 years, with a median age of 14 years and a mean of 13.88 ± 2.27 years (95% CI: 13.16-14.60). The main thoracic Cobb angle ranged from 41° to 55°, with a median of 46° and a mean of 46.98 ± 4.30° (95% CI: 45.62-48.33). The AVVA varied across thoracic levels. At T7, AVVA ranged from 39° to 79°, with a median of 69° and a mean of 67.71 ± 7.50° (95% CI: 65.34-70.08). At T8, values ranged from 41° to 84°, with a median of 63° and a mean of 64.15 ± 7.83° (95% CI: 61.68-66.62). At T9, AVVA ranged from 51° to 90°, with a median of 66° and a mean of 66.24 ± 9.34° (95% CI: 63.30-69.19). At T10, AVVA ranged from 52° to 90°, with a median of 74° and a mean of 73.61 ± 7.43° (95% CI: 71.26-75.96) (Table 1, Figure 1). Overall, Figure 1 demonstrates the progressive change in azygos vein position relative to the thoracic pedicle corridor across vertebral levels T7-T10, highlighting the potential risk of vascular breach during thoracic pedicle screw placement in AIS.

**Table 1:** Distribution of age, Cobb angle, and azygos vein-vertebral angle (AVVA) at thoracic levels T7–T10 in 41 thoracic adolescent idiopathic scoliosis patients

Variables	Minimum to maximum value	Median (Q1–Q3)	Mean ± SD	95% CI for mean
Age (years)	11 to 18	14 (13-16)	13.88 ± 2.27	13.16 to 14.6
Cobb's angle (^o^)	41 to 75	46 (44-50)	46.98 ± 4.3	45.62 to 48.33
Azygos vein-vertebral angle (AVVA) (^o^)				
AVVA (^o^) T7	39 to 79	69 (62-72)	67.71 ± 7.5	65.34 to 70.08
AVVA (^o^) T8	41 to 84	63 (58-68)	64.15 ± 7.83	61.68 to 66.62
AVVA (^o^) T9	51 to 90	66 (60-74)	66.24 ± 9.34	63.3 to 69.19
AVVA (^o^) T10	52 to 90	74 (68-78)	73.61 ± 7.43	71.26 to 75.96

**Figure 1:**
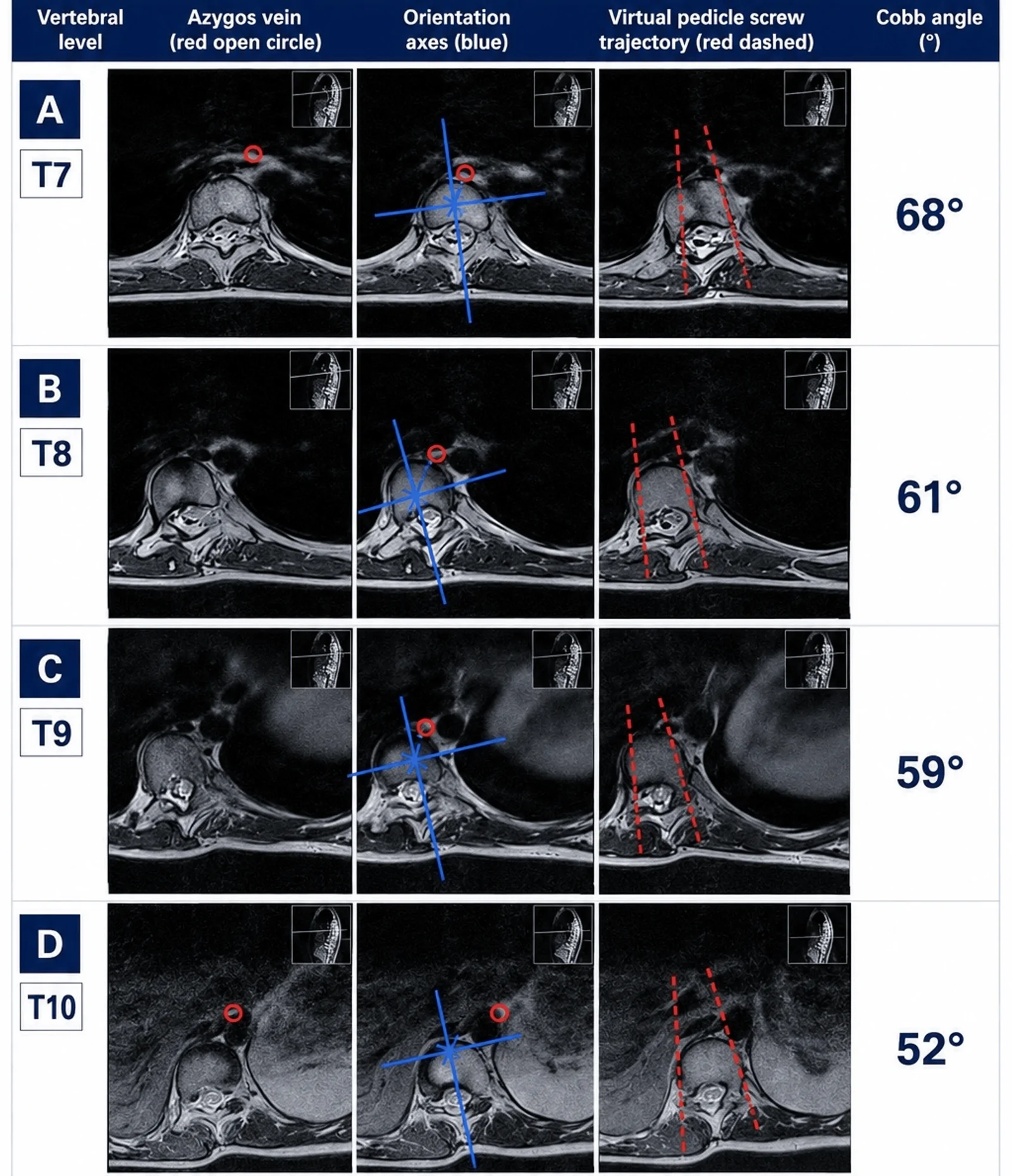
Axial MRI evaluation of azygos vein position and virtual pedicle screw trajectory at thoracic levels T7-T10 in adolescent idiopathic scoliosis (A) Axial MRI image at the digitally annotated T7 vertebral level demonstrating the position of the azygos vein (red open circle) relative to the vertebral body. Blue lines represent vertebral orientation axes, while red dashed lines indicate the virtual pedicle screw trajectory. The Cobb angle was digitally annotated as 68°. (B) Axial MRI image at the digitally annotated T8 vertebral level showing the azygos vein position (red open circle) in relation to the pedicle corridor and planned pedicle screw trajectory. Blue lines indicate vertebral orientation axes, and red dashed lines represent the simulated screw path. The Cobb angle was digitally annotated as 61°. (C) Axial MRI image at the digitally annotated T9 vertebral level illustrating the spatial relationship between the azygos vein (red open circle) and the proposed pedicle screw trajectory. Blue orientation axes and red dashed virtual screw trajectory are shown. The Cobb angle was digitally annotated as 59°. (D) Axial MRI image at the digitally annotated T10 vertebral level demonstrating azygos vein location (red open circle) and the virtual pedicle screw trajectory relative to the vertebral anatomy. Blue lines represent vertebral orientation axes, and red dashed lines represent the simulated screw path. The Cobb angle was digitally annotated as 52°.

Comparison with normal reference values revealed that the mean Cobb angle in cases was slightly lower than the normal value. By contrast, AVVA values were significantly higher in scoliosis patients at all thoracic levels. At T7, the mean AVVA was 67.71° in cases versus 57.9° in normal subjects; at T8, 64.15° versus 50.4°; at T9, 66.24° versus 51°; and at T10, 73.61° versus 54.1° (all p < 0.001) (Table 2).

**Table 2:** Comparison of Cobb angle and azygos vein–vertebral angle (AVVA) between normal reference values and thoracic adolescent idiopathic scoliosis cases †Normal reference values from Jiang et al. [10]. *Statistically significant at p < 0.05.

Variables		Reference value for normal†	Cases (n=41)	Mean difference (95% CI)	Student t test	p- value
Cobb angle (°)		49.0 ± 8.3	46.98 ± 4.30	-2.02 (-3.38 to -0.67)	-3.012	0.004*
Azygos vein–vertebral angle (AVVA) (^o^)	AVVA (°) T7	57.9 ± 11.7	67.71 ± 7.50	9.81 (7.44 to 12.18)	8.368	0.000*
	AVVA (°) T8	50.4 ± 11.7	64.15 ± 7.83	13.75 (11.28 to 16.22)	11.244	0.000*
	AVVA (°) T9	51.0 ± 6.7	66.24 ± 9.34	15.24 (12.30 to 18.19)	10.453	0.000*
	AVVA (°) T10	54.1 ± 9.9	73.61 ± 7.43	19.51 (17.16 to 21.86)	16.807	0.000*

The distribution of cases demonstrating potential azygos vein involvement across thoracic levels is presented in Table 3. The highest frequency was observed at T8, with 37 (90.2%) cases, followed by T7, with 36 (87.8%) cases, and T9, with 33 (80.5%) cases. The lowest frequency was at T10, where 16 (39%) cases demonstrated potential involvement.

**Table 3:** Distribution of thoracic levels showing potential azygos vein involvement among 41 thoracic adolescent idiopathic scoliosis patients A single patient may be counted at multiple thoracic levels if the virtual screw trajectory crossed the azygos vein at more than one level.

Level	Total number N (%)
T7	36 (87.8%)
T8	37 (90.2%)
T9	33 (80.5%)
T10	16 (39%)

No statistically significant correlation was observed between the Cobb angle and AVVA at any thoracic level. Correlation coefficients were weak and negative at T7 (r = −0.040), T9 (r = −0.071), and T10 (r = −0.178), with a negligible positive correlation at T8 (r = 0.004). All associations were non-significant (p > 0.05) (Table 4).

**Table 4:** Correlation between Cobb angle and azygos vein-vertebral angle (AVVA) at thoracic levels (T7-T10) Pearson correlation test. All p-values > 0.05 indicate non-significant correlations. AVVA = azygos vein-vertebral angle

Variables		AVVA (°) T7	AVVA (°) T8	AVVA (°) T9	AVVA (°) T10
Cobbs angle	Correlation coefficient	-0.040	0.004	-0.071	-0.178
	Sig. (2-tailed)	0.803	0.978	0.661	0.264

A virtual pedicle screw trajectory was created for the left and right sides at each level. The azygos vein was considered at risk if the trajectory crossed its path. The AVVA on the concave side (where the azygos vein is typically displaced) was compared with the convex side using an independent t-test. The results are shown in Table 5. Significantly higher AVVA values were observed on the concave side at T8 (mean difference 8.9°, p = 0.003) and T9 (mean difference 11.2°, p = 0.001), indicating greater medial deviation and therefore higher risk of screw penetration on the concave side at these levels. No significant side-to-side differences were observed at T7 (p = 0.21) or T10 (p = 0.18) (Table 5).

**Table 5:** Side-to-side comparison of AVVA (concave vs. convex) and risk of pedicle screw penetration at thoracic levels T7-T10 AVVA = azygos vein–vertebral angle. * shows a significant p-value. Significant p-values (<0.05) indicate a higher risk on the concave side.

Level	AVVA concave side (°) mean ± SD	AVVA convex side (°) mean ± SD	Mean difference (°)	Paired t- test value	p-value
T7	68.2 ± 7.8	67.2 ± 7.4	1.0	1.26	0.21
T8	68.5 ± 8.1	59.6 ± 7.2	8.9	3.05	0.003
T9	71.3 ± 9.2	60.1 ± 8.5	11.2	3.37	0.001*
T10	74.5 ± 7.9	72.7 ± 7.1	1.8	1.35	0.18

## Discussion

The present study evaluated the anatomical displacement of the azygos vein in adolescents with thoracic AIS using magnetic resonance imaging (MRI), with particular attention to its potential relationship with a simulated pedicle screw trajectory. The findings demonstrated that the AVVA at T7-T10 was significantly different from previously reported reference values, indicating altered azygos vein positioning in thoracic AIS compared with the published normative data of Jiang et al. [10]. These findings provide level-specific anatomical information that may be relevant when assessing the relationship between the azygos vein and planned pedicle screw trajectories.

The mean AVVA values in the present cohort ranged from 64.15° at T8 to 73.61° at T10 and were significantly greater than the corresponding values reported in healthy adolescents by Jiang et al. [10]. The altered course of the azygos vein may be related to the three-dimensional deformity characteristic of AIS, which includes vertebral rotation and translation [3,11]. Such deformity can alter the spatial relationships between the vertebral bodies and adjacent mediastinal structures [12]. However, the present study assessed the angular position of the vein rather than the complete three-dimensional anatomy surrounding the intended screw trajectory.

Among the four thoracic levels studied, the highest mean AVVA was observed at T10 (73.61°), followed by T7 (67.71°), T9 (66.24°), and T8 (64.15°). By contrast, the highest frequency of virtual trajectory-azygos vein intersection was observed at T8 (37/41, 90.2%), followed by T7 (36/41, 87.8%) and T9 (33/41, 80.5%), whereas T10 demonstrated the lowest frequency (16/41, 39.0%). The higher frequency of virtual intersection at T8 may be related to the altered three-dimensional vertebral anatomy at the apex of the thoracic curve, which can modify the relationship between the azygos vein and the intended pedicle screw trajectory [13]. Nevertheless, the difference between AVVA magnitude and virtual intersection frequency indicates that a larger AVVA does not necessarily correspond to a higher frequency of trajectory-vein intersection.

This discordance suggests that AVVA describes the angular position of the azygos vein relative to the vertebral body but does not, by itself, capture the complete three-dimensional spatial relationship between the vein and the planned pedicle screw trajectory. Therefore, AVVA should be interpreted as an anatomical descriptor rather than as a standalone predictor of potential trajectory-vein conflict. Patient-specific cross-sectional imaging with assessment or simulation of the planned screw trajectory may provide a more direct evaluation of this spatial relationship [14].

The relationship between the magnitude of the coronal curve and the position of the azygos vein was also examined. No statistically significant linear association was identified between the main thoracic Cobb angle and AVVA at any of the four levels, with correlation coefficients ranging from −0.178 to 0.004. These findings suggest that, within this cohort, greater coronal curve magnitude was not significantly associated with greater AVVA. Jiang et al. reported similar findings, with no or weak correlations at most levels except T9 and T10 [10]. However, the absence of a statistically significant linear association should not be interpreted as evidence that no relationship exists, as the association may be influenced by sample size, nonlinear relationships, vertebral rotation, curve characteristics, apical level, or other anatomical factors not assessed in the present study. Thus, the Cobb angle alone may not adequately characterize the patient-specific spatial relationship between the azygos vein and a planned pedicle screw trajectory, supporting the role of cross-sectional imaging for individualized preoperative anatomical assessment [15].

The side-to-side comparison provides an additional level-specific observation. AVVA differed significantly between the concave and convex sides at T8 (mean difference, 8.9°; p = 0.003) and T9 (mean difference, 11.2°; p = 0.001), whereas no statistically significant difference was observed at T7 or T10. These findings suggest that the position of the azygos vein relative to the vertebral anatomy may differ between the concave and convex sides at selected thoracic levels. Because these measurements were obtained from the same patients, a paired analysis was performed for the side-to-side comparison. Although these differences may be relevant to individualized assessment of the intended screw trajectory, they should not be interpreted as evidence of an increased incidence of vascular injury, as clinical screw placement and vascular outcomes were not evaluated. Consequently, options such as shorter screws or alternative fixation strategies, including hooks or hybrid constructs, should be considered only as potential surgical considerations requiring evaluation in future clinical studies rather than as established recommendations based on the present findings [16].

The potential clinical relevance of these anatomical findings lies in the possibility that an intended pedicle screw trajectory may be in proximity to the azygos vein. Although iatrogenic azygos vein injury during thoracic pedicle screw placement is uncommon, vascular injury may result in clinically significant complications such as hemorrhage and hematoma [17]. Venous injury may also have delayed manifestations, including pseudoaneurysm or venous thrombosis [18]. Nevertheless, the present study did not evaluate actual screw insertion, intraoperative vascular injury, postoperative hemorrhage, hematoma, pseudoaneurysm, venous thrombosis, or other clinical complications. Therefore, the virtual trajectory-vein intersections identified in this study should be regarded as potential anatomical conflicts rather than confirmed vascular injuries. The findings may support consideration of patient-specific cross-sectional imaging during preoperative planning, particularly when the planned trajectory appears to approach the azygos vein. Still, prospective clinical studies are required to establish whether these anatomical findings translate into clinically meaningful complications.

A strength of this study is the level-specific assessment of the azygos vein from T7 to T10 in patients with thoracic AIS. The use of complementary measures, AVVA, virtual trajectory-vein intersection, side-to-side comparison, and assessment of the relationship between AVVA and Cobb angle, provides a broader assessment of the anatomical relationship than reliance on a single angular measurement. In particular, the difference between the level with the highest AVVA and the level with the highest virtual trajectory-vein intersection highlights the importance of considering the intended trajectory rather than relying solely on AVVA magnitude.

This study has several limitations that should be considered when interpreting the findings. First, the cross-sectional design provides an assessment of azygos vein position at a single time point. It cannot determine how AVVA or the spatial relationship between the azygos vein and the intended screw trajectory changes over the course of curve progression, skeletal maturation, or treatment. Second, although an a priori sample-size calculation based on the standard deviation and precision reported by Jiang et al. [10] indicated a minimum required sample size of 36 participants, 41 patients were ultimately included; the final sample remained modest. This may have limited the statistical power to detect weaker associations and reduced the precision of analyses involving additional anatomical factors.

Third, the comparison was made with previously published normative values rather than with a contemporaneous control group. Differences between the present cohort and the reference population in age, demographic characteristics, MRI acquisition protocols, patient positioning, image orientation, and measurement techniques may have introduced heterogeneity. They should be considered when interpreting the observed differences in AVVA. Fourth, the study did not assess measurement reliability or intra-observer or inter-observer variability. Future studies should incorporate standardized measurement protocols and assess reproducibility.

Fifth, the virtual pedicle screw trajectory-azygos vein intersection was used as a surrogate anatomical outcome and has not been validated in the present study against actual intraoperative screw placement or postoperative vascular complications. The simulation also does not account for all factors that may influence the final clinical trajectory, including intraoperative positioning, screw diameter and length, three-dimensional screw orientation, surgical technique, and surgeon-specific adjustments. Accordingly, the observed intersections should not be interpreted as evidence of confirmed vascular injury or as a direct estimate of the risk of clinical complications.

Sixth, the distribution of the curve apex or apical vertebral level was not specifically analyzed in relation to AVVA or virtual trajectory-vein intersection. Other potentially relevant anatomical factors, including vertebral rotation, sagittal alignment, thoracic kyphosis, and detailed three-dimensional deformity characteristics, were also not incorporated into the analysis. Seventh, repeated measurements at T7-T10 were obtained from the same patients, and the analyses did not specifically account for within-patient clustering across vertebral levels. In addition, multiple level-specific and side-to-side comparisons were performed without adjustment for multiple testing, which increases the possibility of type I error.

Finally, the absence of a statistically significant linear association between Cobb angle and AVVA should not be interpreted as proof that no relationship exists. The possibility of nonlinear associations or relationships influenced by other anatomical characteristics cannot be excluded. These limitations indicate that the present findings should be regarded primarily as level-specific anatomical observations and hypothesis-generating evidence. Larger, prospective, multicenter studies incorporating standardized MRI protocols, reliability assessment, three-dimensional trajectory analysis, and correlation with actual surgical and postoperative vascular outcomes are required to determine the clinical significance of the observed trajectory-vein relationships.

## Conclusions

This study demonstrated significant level-specific differences in the AVVA at T7-T10 in adolescents with thoracic AIS compared with previously published reference values. The highest mean AVVA was observed at T10. By contrast, the highest frequency of virtual trajectory-azygos vein intersection occurred at T8, indicating that AVVA magnitude alone does not fully represent the three-dimensional spatial relationship between the azygos vein and a planned pedicle screw trajectory. No statistically significant linear association was identified between the main thoracic Cobb angle and AVVA at T7-T10, suggesting that coronal curve magnitude alone may not adequately characterize this anatomical relationship. The observed side-to-side differences at T8 and T9 further demonstrate level-specific variation in azygos vein position.

Patient-specific preoperative cross-sectional imaging with assessment of the intended pedicle screw trajectory may therefore provide useful anatomical information for individualized surgical planning. However, because actual pedicle screw placement and intraoperative or postoperative vascular outcomes were not evaluated, the virtual trajectory-azygos vein intersections should be interpreted as potential anatomical conflicts rather than confirmed vascular injuries. Further prospective studies incorporating standardized imaging, three-dimensional trajectory assessment, and correlation with surgical and postoperative vascular outcomes are required to establish the clinical significance of these findings.
